# Novel Enzyme-Assisted Recycle Amplification Strategy for Tetracycline Detection Based on Oxidized Single-Walled Carbon Nanohorns

**DOI:** 10.3390/molecules29071444

**Published:** 2024-03-23

**Authors:** Tingting Feng, Shuzhu Yan, Yu Huang

**Affiliations:** College of Traditional Chinese Medicine and Food Engineering, Shanxi University of Chinese Medicine, Jinzhong 030619, China; 15034578921@163.com (S.Y.); 18835740331@163.com (Y.H.)

**Keywords:** oxSWCNHs, cryonase enzyme, signal amplification, tetracycline

## Abstract

In this study, oxidized single-walled carbon nanohorns (oxSWCNHs) were prepared using nitric acid oxidation and subsequently combined with 3′6-carboxyfluorescein through charge transfer to prepare fluorescent probes. These oxSWCNHs were used to quench fluorogen signals at short distances and dissociate ssDNA using cryonase enzymes. We established a method for rapidly detecting tetracycline (TC) in complex samples based on the amplification of cryonase enzyme signals. After optimizing the experimental conditions, our method showed a detection limit of 5.05 ng/mL, with good specificity. This method was used to determine the TC content in complex samples, yielding a recovery rate of 90.0–103.3%. This result validated the efficacy of our method in detecting TC content within complex samples.

## 1. Introduction

Tetracycline (TC) [1] is synthesized by first extracting chlortetracycline from Streptomyces aureofaciens culture medium and then removing the chlorine atom in the catalytic chlorination of chlortetracycline [2,3]. Our commonly used TC antibiotics belong to a class of broad-spectrum antibiotics. In addition to their antibacterial effect on gram-positive (G+) and gram-negative (G−) bacteria, they are widely used to combat infections caused by rickettsia, mycoplasma, chlamydia, and spirochetes, among others. They exhibit a superior effect on gram-negative bacteria, possessing bacteria-killing properties at high concentrations [4,5]. In addition, TC is extensively employed as an antibiotic in animal rearing and is frequently detected in municipal wastewater, surface water, groundwater, and even drinking water [4,5,6]. If repeatedly ingested, it can cause colony disorders, drug resistance, dental health complications, and other adverse reactions in humans. Therefore, detecting its presence is imperative. Currently, various methods have been developed for TC detection. Traditional methods, such as HPLC [7], electrochemical [8], chromatography–mass spectrometry [9], and immunoassays [10], are well established. Although the first three methods are accurate and reliable, expensive equipment and cumbersome operations pose challenges, which limits their suitability for large-scale sample screening and rapid field detection, making it difficult to promote their widespread use, particularly at the grassroots level. Immunoassays are a common fast screening method for TC detection, among which enzyme-linked immunosorbent assays and the colloidal gold strip method are the most prevalent. However, these methods are limited in rapid detection due to their lengthy antibody preparation cycle and high costs. Therefore, developing a simple and sensitive detection technology for TC is crucial.

Fluorescence resonance energy transfer is a radiation energy transfer process, which is widely used in fluorescence biosensors. In light excitation, the excited state donor can transfer energy to the ground state receptor through dipole–dipole coupling. As a highly distance-dependent process, the distance between two fluorophores is usually less than 10 nm, and the donor’s emission spectrum and the acceptor’s absorption spectrum overlap well [11,12]. FRET has the advantages of fast analysis, high sensitivity, good selectivity, and little or no pollution. At present, it has been widely used to study through nucleic acid analysis [13], immunoassay [14], and protein analysis. In this paper, a simple and rapid fluorescence sensing method for TC detection based on aptamer fluorescence energy resonance transfer is proposed. This method is rapid, simple, and has a low cost, and has been successfully applied to the detection of TC in cosmetic samples with satisfactory results. In theory, this approach can be applied to all nucleic acid aptamers, regardless of their size or structural properties, and is particularly suitable for targets with long sequence nucleic acid aptamers.

**Scheme 1:**
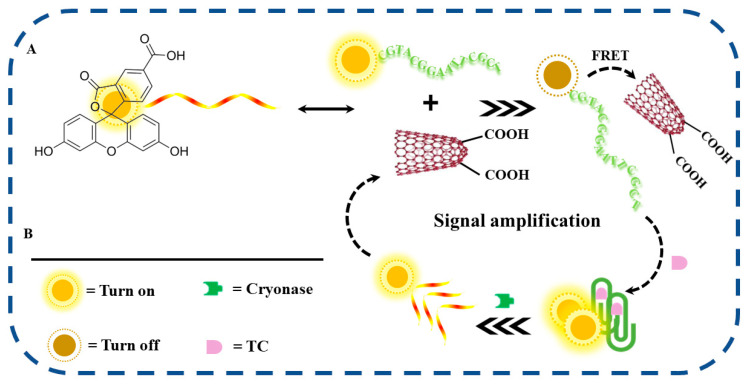
Schematic illustration of the oxidized single-walled carbon nanohorn (oxSWCNHs)-based fluorescent aptasensor for tetracycline (TC) assay. (**A**) Changes in fluorescence in the presence and absence of the target tetracycline (**B**) Interpretation of the figure represented in (**A**).

Signal amplification, a common method for improving sensitivity in detection analysis, primarily relies on the cyclic interaction between a target and different nucleic acid signal probes, with the output signal amplified under the action of the target at the same concentration [15,16]. Nucleases can recognize and cut specific base sequences or functional groups, catalyzing the hydrolysis of phosphodiester bonds between nucleotides. For example, cryonase, DNase I, RNase H, endonuclease IV, and Exo III are often used as cutting enzymes to promote target circulation [17]. Cryonase exhibits high enzymatic activity and can act on all DNA and RNA types (single, double, linear, and circular). Thus, we utilized the catalytic enzymatic hydrolysis of ssDNA by cryonase to reduce background output signals and relatively amplify detection signals. Using ssDNA as a substrate and the nucleic acid dye FAM as the fluorescence signal, we propose the label-free detection of TC using a fluorescence aptamer sensor based on cryonase-assisted signal amplification. Furthermore, we investigated its detection ability in complex samples. The experimental principle is shown in Scheme 1. When the target TC is absent, oxSWCNHs—serving as regulatory factors—adsorb the aptamer chain through a π–π packing interaction and display a significantly low fluorescence signal. After adding the target TC, the aptamer specifically binds to the target and dissociates from the surface of the oxSWCNHs. Simultaneously, cryonase degrades the aptamer chain, releasing fluorescent molecules and allowing the target TC to enter the next cycle, thereby generating strong fluorescence signals. Thus, the TC concentration can be analyzed by detecting changes in system fluorescence intensity.

## 2. Results and Discussion

### 2.1. Feasibility of the Proposed Method

**Figure 1:**
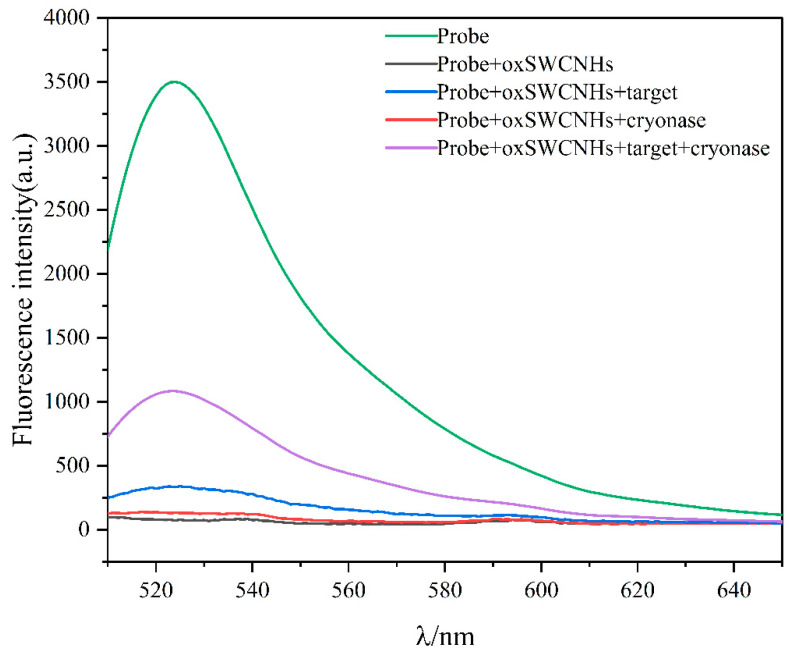
Method feasibility analysis in 20 mmol/L Tris—HCl buffer (5 mmol/L MgCl_2_, 100 mmol/L NaCl, pH 8.0). FAM-aptamer: 5 nmol/L, oxSWCNHs: 500 ng/mL, cryonase: 1.0 mU/μL, TC: 17 ng/mL.

In this study, we designed a fluorescence-based method for the sensitive detection of TC using cryonase-assisted signal amplification. As shown in Figure 1, the design strategy involved using TC-aptamer as the recognition unit and FAM as the fluorescence probe. When the FAM-labeled aptamer was excited at a certain wavelength, fluorescence was observed at 525 nm (green line). In the presence of oxSWCNHs, aptamers adsorbed on the surface of oxSWCNHs shortened the distance between FAM and oxSWCNHs. This induced a fluorescence resonance energy transfer effect between FAM and oxSWCNHs, and the fluorescence of FAM was quenched (black line), serving as the quencher in this study. The oxSWCNHs effectively quenched approximately 98% of FAM fluorescence. Introducing cryonase to the GO/aptamer induced minimal changes in the fluorescence signal, indicating that oxSWCNHs can protect the aptamer from cryonase-induced degradation (red line). The fluorescence of FAM was recovered after TC was added, showing increased fluorescence intensity compared with the absence of TC (blue line). The strong affinity between TC and the aptamer was crucial in this process, resulting in the TC-aptamer complex preventing the adsorption of the FAM-labeled aptamer onto the oxSWCNHs surface. In addition, the fluorescent intensity significantly increased with the presence of cryonase in the system (purple line), displaying a signal strength N times that of traditional strategies lacking enzyme amplification. This phenomenon is due to the cryonase catalytic complex being destroyed, which results in the target TC being released. The released TC combines with another aptamer, leading to the recycling of the TC target and signal amplification. These results validate the feasibility of our method, which involves measuring the TC concentration by monitoring changes in fluorescence intensity in the aptamer sensing platform.

### 2.2. Characterization of oxSWCNHs

**Figure 2:**
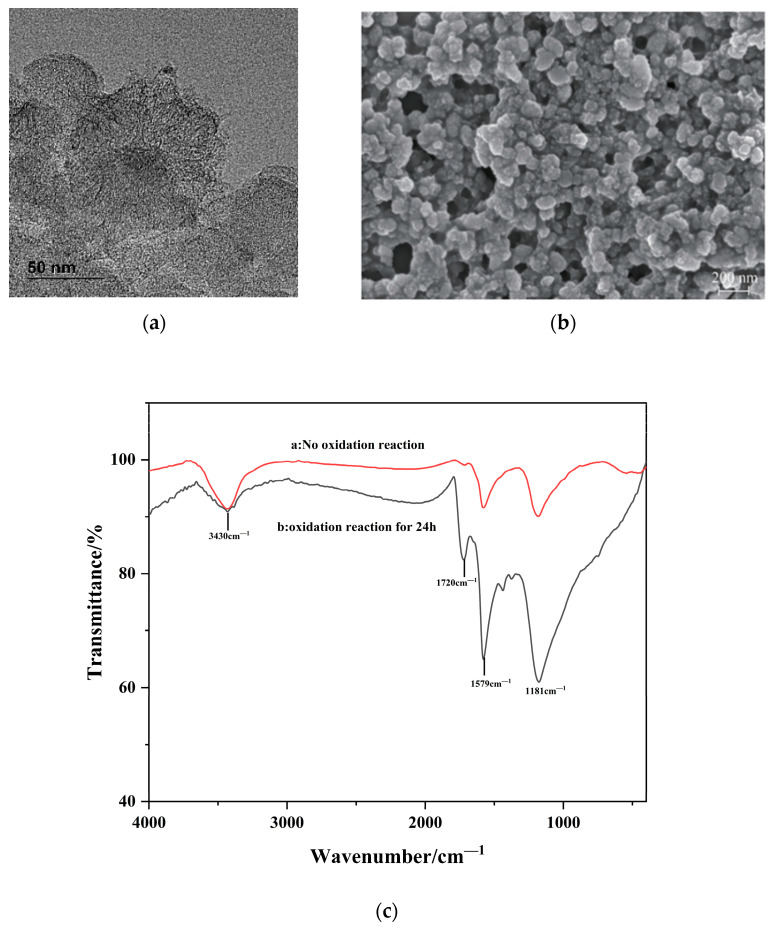
(**a**) Transmission electron microscopy image of oxSWCNHs. (**b**) Scanning electron microscopy image of oxSWCNHs. (**c**) IR spectra of the oxSWCNHs composite.

To construct the oxSWCNHs/adapter fluorescent biosensor, we prepared and characterized oxSWCNHs. The transmission electron microscopy and scanning electron microscopy results (Figure 2a,b) showed structural changes in oxSWCNHs compared with SWCNHs, with the aggregates in the original structure exhibiting good dispersion, which enhanced their hydrophilicity. The potential value of oxSWCNHs after oxidation was determined to be −33.35 ± 1.06 (mv), indicating an increase after oxidation. The results showed that SWCNHs obtained through nitric acid oxidation may contain hydrophilic groups, such as -COOH and -OH. To further determine the structure of oxSWCNHs (Figure 2c), the chemical groups of oxSWCNHs were determined using Fourier transform near-infrared spectroscopy. The broad NIR peak at 3440 cm^−1^ indicated -OH in oxSWCNHs–COOH, whereas the NIR peak at 1730 cm^−1^ indicated -C=O in -COOH. This showed that the -COOH on the oxSWCNHs surface increased 24 h after the oxidation reaction, consistent with the observed change in the potential value.

### 2.3. Construction of the Sensing System

**Figure 3:**
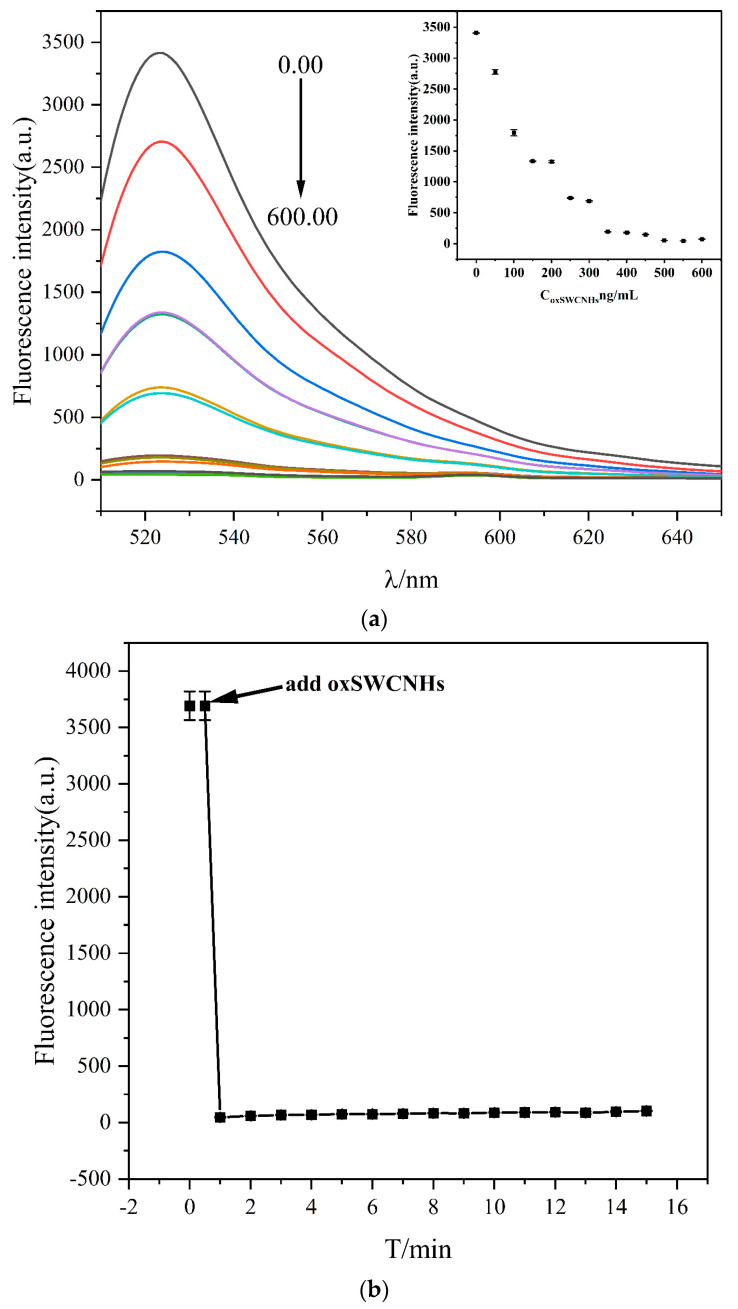
(**a**) Fluorescence intensity of the FAM-aptamer in the presence of oxSWCNHs at different concentrations. oxSWCNHs concentrations from top to bottom are 0, 50, 100, 150, 200, 250, 300, 350, 400, 450, 500, 550, and 600 ng/mL. Other conditions: in 20 mmol/L Tris-HCl buffer (5 mmol/L MgCl_2_, 100 mmol/L NaCl, pH 8.0), FAM-aptamer: 5 nmol/L. (**b**) Effect of reaction time on the fluorescence intensity of the FAM-aptamer in the presence of optimal oxSWCNHs. Other conditions: in 20 mmol/L Tris-HCl buffer (5 mmol/L MgCl_2_, 100 mmol/L NaCl, pH 8.0), FAM-aptamer: 5 nmol/L, oxSWCNHs: 500 ng/mL.

To improve the performance of the aptamer sensor, we optimized the experimental conditions and investigated the effects of the oxSWCNHs concentration, quenching time, cryonase concentration, and enzyme digestion time on the system. We first investigated the effect of the oxSWCNHs mass concentration on system fluorescence intensity (0, 50, 100, 150, 200, 250, 300, 350, 400, 450, 500, 550, and 600 ng/mL). As shown in Figure 3a, the fluorescence intensity decreased with increasing oxSWCNHs mass concentrations, reaching optimal levels at 500 ng/mL. Additionally, the oxSWCNHs quenching time influenced the experimental process. As time progressed, the reaction time reached a plateau after 10 min, identifying the optimal quenching time as 10 min (Figure 3b).

**Figure 4:**
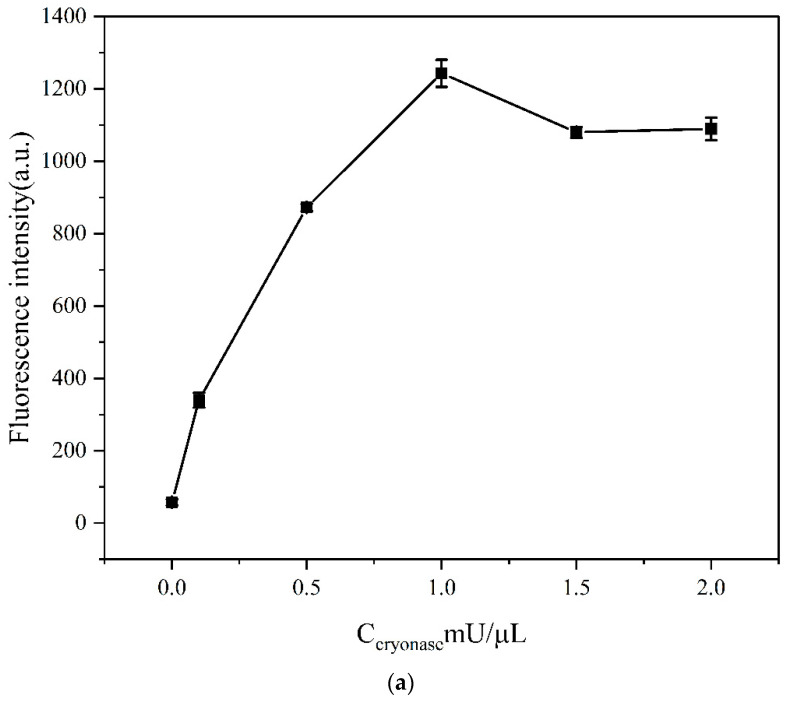

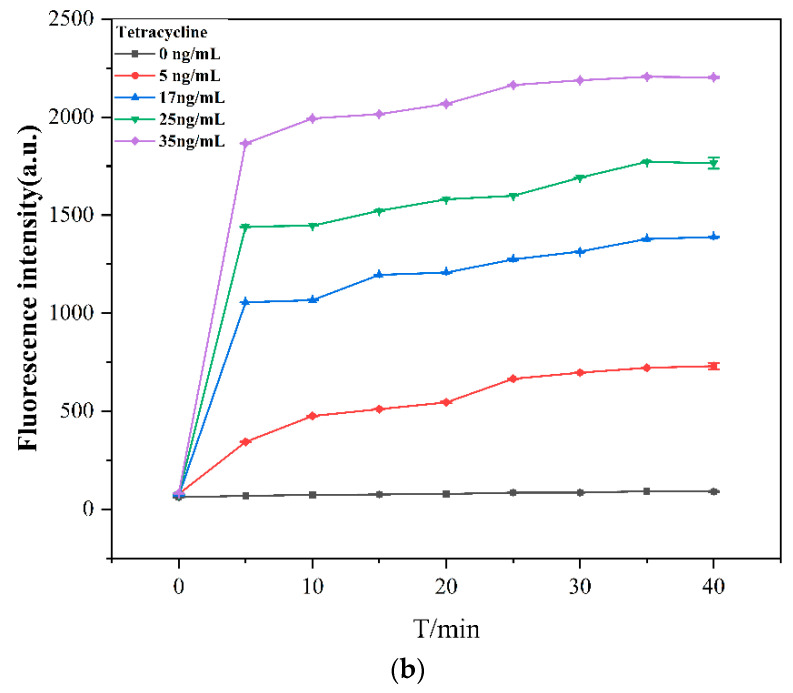
(**a**) Fluorescence intensity of the FAM-aptamer in the presence of varying cryonase concentrations (0, 0.2, 0.5, 1.0, 1.5, and 2.0 mU/μL). Other conditions: in 20 mmol/L Tris-HCl buffer (5 mmol/L MgCl_2_, 100 mmol/L NaCl, pH 8.0), FAM-aptamer: 5 nmol/L, oxSWCNHs: 500 ng/mL, TC:17 ng/mL. (**b**) Trend of the fluorescence intensity of the FAM-aptamer with time at fixed cryonase concentrations (0, 5, 10, 15, 20, 25, 30, 35, and 40 min). Other conditions: in 20 mmol/L Tris-HCl buffer (5 mmol/L MgCl_2_, 100 mmol/L NaCl, pH 8.0); FAM-aptamer: 5 nmol/L; oxSWCNHs: 500 ng/mL; cryonase: 1.0 mU/μL; TC: 0, 5, 17, 25, and 35 ng/mL.

The enzymatic cleavage cycle is crucial for signal amplification. As shown in Figure 4a, low cryonase activity led to the incomplete hydrolysis of aptamers detached from the surface of oxSWCNHs, resulting in a low fluorescence signal. However, increasing cryonase activity (0, 0.2, 0.5, 1.0, 1.5, 2.0 mU/μL) led to more aptamers being hydrolyzed and a significant increase in fluorescence intensity. At 1 mU/μL cryonase activity, maximum fluorescence intensity was attained; therefore, the cryonase activity was set at 1 mU/μL in subsequent experiments. The enzyme reaction time, an important condition in the experiment, was also evaluated. A decisive reaction time yields an incomplete enzyme digestion reaction; however, a lengthy reaction time will affect the entire experiment. Following cryonase addition, the system reacted for 0, 5, 10, 15, 20, 25, 30, 35, and 40 min (Figure 4b) and the measured fluorescence intensity was recorded. Fluorescence intensity increased sharply in the initial stage and gradually stabilized when the reaction time was extended to 40 min. We speculated that the specificity between the FAM-modified aptamer and the target molecule reached saturation at 30 min. Therefore, the optimal action time for cryonase was selected as 30 min.

### 2.4. General Procedure for the Tetracycline Assay

**Figure 5:**
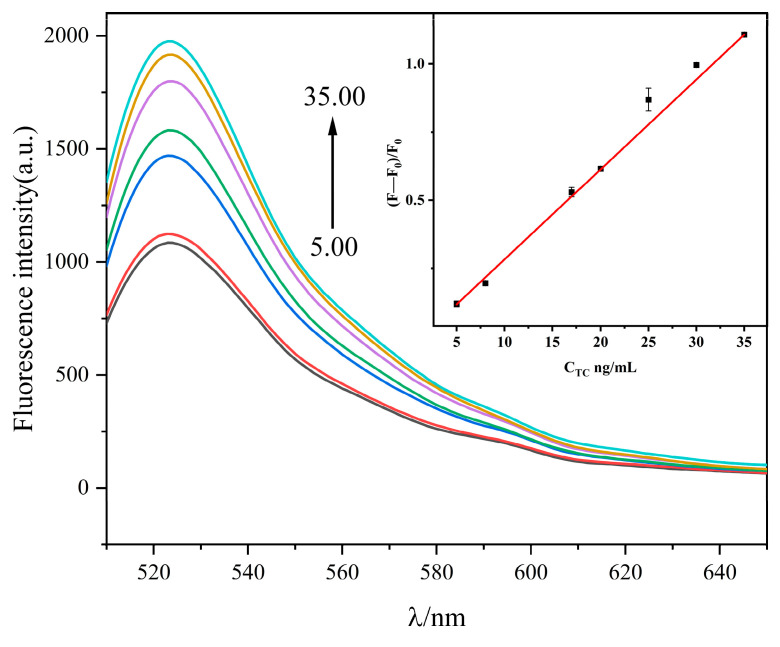
Emission spectra of the FAM-aptamer when different TC concentrations (5, 8, 17, 20, 25, 30, and 35 ng/mL) were added. Illustration of the linear calibration curve of the aptamer fluorescence sensor. F_0_ and F represent the fluorescence intensity of the system in the absence and presence of TC. Other conditions: in 20 mmol/L Tris-HCl buffer (5 mmol/L MgCl_2_, 100 mmol/L NaCl, pH 8.0), FAM-aptamer: 5 nmol/L, oxSWCNHs: 500 ng/mL, cryonase: 1.0 mU/μL.

Under these optimized conditions, the detection performance of the oxSWCNHs/aptamer/cryonase system was analyzed. Figure 5 shows changes in the fluorescence emission spectra of TC across various concentration gradients upon addition to the oxSWCNHs/aptamer/cryonase system. With increasing TC concentrations, the system fluorescence intensity increased regularly. A good linear relationship exists between the (F − F_0_)/F_0_ value and the TC concentration (5–35 ng/mL) (Figure 5). The linear equation (F − F_0_)/F_0_ = 0.0343[TC]–0.0593, with a correlation coefficient of R^2^ = 0.9951, demonstrates the good quantitative detection performance of the sensor. Calculating based on the 3σ/k rule, the detection limit of TC was 5.05 ng/mL, proving that our proposed method meets the requirements of TC sample detection. A comparison with existing TC detection methods (Table 1) highlighted the measurement method of signal amplification as less time-consuming and possessing improved sensitivity.

### 2.5. Specific Study

**Figure 6:**
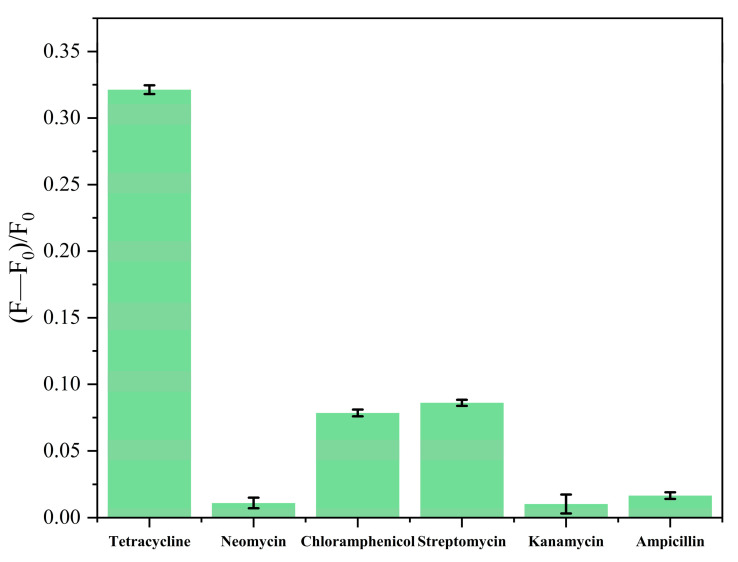
Selective determination of TC and 100 ng/mL of other potentially interfering substances, including neomycin, chloramphenicol, streptomycin, kanamycin, and ampicillin. Other conditions: in 20 mmol/L Tris-HCl buffer (5 mmol/L MgCl_2_, 100 mmol/L NaCl, pH 8.0), FAM-aptamer: 5 nmol/L, oxSWCNHs: 500 ng/mL, cryonase: 1.0 mU/μL.

Six commonly used antibiotics, including tetracycline, kanamycin, streptomycin, neomycin, ampicillin, and chloramphenicol, were selected as analytes to evaluate the selectivity of the fluorescence sensor to TC by measuring changes in fluorescence intensity (F − F_0_)/F_0_. As shown in Figure 6, only TC caused significant fluorescence enhancement of the system, whereas other antibiotics caused only a smaller fluorescent signal. The sensitive response of this fluorescent biosensor to TC provides good selectivity compared to several antibiotics.

### 2.6. Sensor Stability

**Figure 7:**
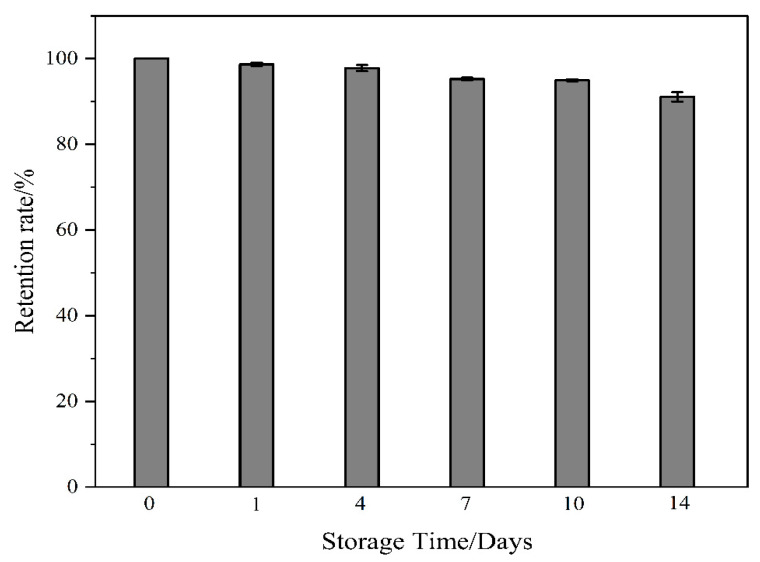
Research on sensor stability. Other conditions: in 20 mmol/L Tris-HCl buffer (5 mmol/L MgCl_2_, 100 mmol/L NaCl, pH 8.0), FAM-aptamer: 5 nmol/L, oxSWCNHs: 500 ng/mL, cryonase: 1.0 mU/μL, TC: 25 ng/mL.

To assess whether the sensor can meet long-term detection requirements, a TC concentration of 25 ng/mL was selected and the prepared sensor was stored at 4 °C. The stability of the sensor was tested on Days 1, 4, 7, 10, and 14. The experimental results (Figure 7) revealed that by Day 14, the fluorescence retention rate had reached 90.07%, indicating good stability.

### 2.7. Actual Sample Application

To further evaluate the usefulness of the sensor in complex environments, recovery tests were conducted in sample solutions with varying TC concentrations (8, 20, 25, and 35 ng/mL). As shown in Table 2, the average recovery ranged from 90.00% to 103.31%, and the relative standard deviation (RSD) ranged from 0.31% to 3.79%. The recovery rate and RSD results indicate that the FAM-aptamer, as the biosensing system of a fluorescent probe, holds practical value in TC detection within real samples.

## 3. Materials and Methods

### 3.1. Reagents and Chemicals

The FAM-labeled aptamer [22] (5′-CGT ACG GAA TTC GCT-FAM-3′) was supplied by Hema Biology Co., Ltd. (Hangzhou, China). Single-walled carbon nanohorns (SWCNHs) were purchased from Nanjing Xianfeng Nanomaterial Technology Co., Ltd. (Nanjing, China). Tris(hydroxymethyl)aminomethane, HCl, NaCl, MgCl_2_, tetracycline, ampicillin, kanamycin, chloramphenicol, streptomycin sulfate, and neomycin sulfate were purchased from Maikelin Biology Co., Ltd. (Shanghai, China). Cryonase was obtained from Takara Biology Co., Ltd. (Beijing, China).

### 3.2. Insrumentation

The fluorescence spectra were captured using an RF-6000 fluorescence spectrophotometer (Shimazu, Kyoto, Japan) with an excitation wavelength of 492 nm and an emission wavelength ranging from 510 to 650 nm. Both excitation and emission had bandwidths of 5 nm. A pHS-3E pH meter (Yoke, Shanghai, China) was used for the pH measurements, while a PR124ZH/E electronic balance (Ohaus, Changzhou, China) was used for weighing. An MTH-100 constant-temperature mixing instrument (Hangzhou Miu Instruments Co., Ltd., Hangzhou, China) was used for incubation baths.

### 3.3. Synthesis of oxSWCNHs

Initially, 0.0040 g of dry powder SWCNHs was accurately weighed and placed in a round-bottom flask containing 2 mL of DMF and 2 mL of 30% nitric acid for a 24-h reflux reaction. The resulting liquid underwent centrifugation in a 2-mL centrifuge tube for 4 min (12,000 rpm). The upper yellow liquid was removed and the lower black solid was washed at least five times with distilled water until the reaction liquid reached a neutral pH, yielding the oxSWCNHs solution. The obtained solution was freeze-dried and stored for future use.

### 3.4. TC Detection with oxSWCNHs-FAM Aptasensor

First, a 15 μL 100 nmol/L ssDNA solution was mixed with TC solution at varying concentrations (5, 8, 17, 20, 25, 30, and 35 ng/mL) and incubated at 37 °C for 30 min. Second, 1.5 μL of 100 μg/mL oxSWCNHs was added and allowed to react at room temperature for 10 min. Subsequently, 10 μL of 0.03 U/μL cryonase was added, and incubation continued for another 30 min after thorough mixing. The volume was then adjusted to 300 μL with 20 mmol/L Tris-HCl buffer (5 mmol/L MgCl_2_, 100 mmol/L NaCl, pH 8.0). The fluorescence intensity of the sample was measured at 492 nm, and the collection range was determined to be 510–650 nm. The excitation and emission slits were set at 5 nm. All procedures were conducted under light-avoidant conditions.

### 3.5. Specificity Study

To explore the specificity of the protocol for TC detection, we selected kanamycin, streptomycin, neomycin, ampicillin, and chloramphenicol at concentrations of 200 ng/mL. The determination was conducted following the same method and conditions used for TC detection in Section 2.4.

### 3.6. Determination of Tetracycline in Sample Solution

To evaluate the feasibility of the proposed method, the TC activity in running water, eggs, and milk was analyzed using the standard addition method. Before detection, the running water samples were boiled in an electric furnace for 15 min and filtered with a 0.22-μm micron membrane. Egg samples weighing 1 g were treated with 2 mL of 1% trichloroethylene acid, following the abovementioned steps.

Milk samples weighing 1 g were treated with 1 mL of 1% trichloroacetic acid. Ultrasonic extraction was performed for 20 min, followed by centrifugation at 10,000 r/min for 5 min. The resulting supernatant was filtered using filter paper and adjusted to 5 mL with water before filtration through a 0.22-μm filter membrane to obtain the sample solution.

Different concentrations of TC were spiked into the sample solution. The reaction solution was incubated at 37 °C for 30 min, and the fluorescence intensity was measured at 525 nm with excitation at 492 nm.

## 4. Conclusions

In summary, we developed a convenient and sensitive oxSWCNHs-based TC fluorescence detection method by utilizing the specific recognition ability and enzymatic catalytic activity of cryonase. FAM fluorescent dye was used as a fluorescent signal transmission agent to realize the detection of TC. ssDNA can be hydrolyzed by cryonase, which reduces the fluorescence background signal and relatively amplifies the detection signal to improve the detection sensitivity. Compared with previously reported tetracycline detection strategies, our method demonstrates high selectivity. Notably, this approach detects TC without a complex process and establishes a wide linear range, spanning 5–35 ng/mL. Furthermore, the proposed sensor exhibits potential for developing new approaches to detect other antibiotics.

## Figures and Tables

**Table 1:** Comparison of this method with other reported tetracycline detection methods.

Method	Linear Range	Detection Limit (nmol/L)	Reference
Electrochemical method	1–200 μM	30	[18]
A stick-like intelligent multicolor nano-sensor	25 nM–20 μM	8.7	[19]
Zinc ion-based switch-on Fluorescence-sensing probes	15–300 nM	7	[20]
GO-aptamer	33–267 nM	54.0	[21]
Signal amplification	0–35 ng/mL	2.24	This work

**Table 2:** Recovery rate of tetracycline in the sample solution.

Sample	Added (ng/mL)	Found (ng/mL)	Recovery (%)	RSD (%, *n* = 3)
Tap water	8	7.20	90.00	1.41
	20	19.70	98.50	0.97
	25	24.92	99.68	0.59
	35	36.16	103.31	1.25
Eggs	8	7.52	96.63	1.39
	20	19.89	99.45	3.79
	25	25.31	101.8	0.31
	35	34.35	98.14	0.87
Milk	8	7.55	94.37	1.75
	20	20.20	101.00	2.57
	25	25.31	101.2	0.65
	35	36.02	102.91	3.35

## Data Availability

The data presented in this study are available in article.

## References

[1] Li Q., Jiang L., Li Y., Wang X., Zhao L., Huang P., Chen D., Wang J. (2023). Enhancement of Visible-Light Photocatalytic Degradation of Tetracycline by Co-Doped TiO_2_ Templated by Waste Tobacco Stem Silk. Molecules.

[2] Rawal S., Rawal Y. (2001). Non-antimicrobial properties of tetracyclines--dental and medical implications. West Indian Med. J..

[3] Wang T., Mei Q., Tao Z., Wu H., Zhao M., Wang S., Liu Y. (2020). A smartphone-integrated ratiometric fluorescence sensing platform for visual and quantitative point-of-care testing of tetracycline. Biosens. Bioelectron..

[4] Shen C., Wang M., Xiong M., Zhang Y., Xu C., Ma C., Liu Y., Wang H., Li F. (2021). Selective adsorption and fluorescence sensing of tetracycline by Zn-mediated chitosan non-woven fabric. J. Colloid. Interface Sci..

[5] Xu J., Zhang B., Jia L., Bi N., Zhao T. (2020). Metal-enhanced fluorescence detection and degradation of tetracycline by silver nanoparticle-encapsulated halloysite nano-lumen. J. Hazard. Mater..

[6] Zhang Y., Boyd S.A., Teppen B.J., Tiedje J.M., Zhang W., Zhu D., Li H. (2018). Bioavailability of tetracycline to antibiotic resistant Escherichia coli in water-clay systems. Environ. Poll. (Barking Essex 1987).

[7] Nebot C., Cardelle-Cobas A., García-Presedo I., Patyra E., Cepeda A., Franco C.M. (2022). Identification and Quantification of 29 Active Substances by HPLC-ESI-MS/MS in Lyophilized Swine Manure Samples. Molecules.

[8] Oka H., Ito Y., Ikai Y., Kagami T., Harada K. (1998). Mass spectrometric analysis of tetracycline antibiotics in foods. J. Chromatogr. A.

[9] Hu F., Luo W., Liu C., Dai H., Xu X., Yue Q., Xu L., Xu G., Jian Y., Peng X. (2021). Fabrication of graphitic carbon nitride functionalized P-CoFe_2_O_4_ for the removal of tetracycline under visible light: Optimization, degradation pathways and mechanism evaluation. Chemosphere.

[10] Sivakumar R., Lee N.Y. (2021). Recent progress in smartphone-based techniques for food safety and the detection of heavy metal ions in environmental water. Chemosphere.

[11] Xuan F., Luo X., Hsing I.M. (2013). Conformation-dependent exonuclease III activity mediated by metal ions reshuffling on thymine-rich DNA duplexes for an ultrasensitive electrochemical method for Hg^2+^ detection. Anal. Chem..

[12] Wolfbeis O.S. (2013). Probes, sensors, and labels: Why is real progress slow?. Angew. Chem. Int. Ed..

[13] Xu Q., Cao A., Zhang L.F., Zhang C.Y. (2012). Rapid and label-free monitoring of exonuclease III-assisted target recycling amplification. Anal. Chem..

[14] Sedgwick A.C., Wu L.L., Han H.H., Bull S.D., He X.P., James T.D., Sessler J.L., Tang B.Z., Tian H., Yoon J. (2018). Excited-state intramolecular proton-transfer (ESIPT) based fluorescence sensors and imaging agents. Chem. Soc. Rev..

[15] Guo J.J., Mingoes C., Qiu X., Hildebrandt N. (2019). Simple, Amplified, and Multiplexed Detection of MicroRNAs Using Time-Gated FRET and Hybridization Chain Reaction. Anal. Chem..

[16] Annio G., Jennings T.L., Tagit O., Hildebrandt N. (2018). Sensitivity Enhancement of Förster Resonance Energy Transfer Immunoassays by Multiple Antibody Conjugation on Quantum Dots. Bioconjugate Chem..

[17] Park S.H., Ko W., Lee H.S., Shin I. (2019). Analysis of Protein-Protein Interaction in a Single Live Cell by Using a FRET System Based on Genetic Code Expansion Technology. J. Am. Chem. Soc..

[18] Xu H., Zhang D., Weng X., Wang D., Cai D. (2022). Electrochemically reduced graphene oxide/Cu-MOF/Pt nanoparticles composites as a high-performance sensing platform for sensitive detection of tetracycline. Mikrochim. Acta..

[19] Jia L., Chen R., Xu J., Zhang L., Chen X., Bi N., Gou J., Zhao T. (2021). A stick-like intelligent multicolor nano-sensor for the detection of tetracycline: The integration of nano-clay and carbon dots. J. Hazard. Mater..

[20] Zhan Y.C., Tsai J.J., Chen Y.C. (2022). Zinc Ion-Based Switch-on Fluorescence-Sensing Probes for the Detection of Tetracycline. Molecules.

[21] Li L.S., Liang H.W., Wu C.T., Li S., Zhang Y.X., Song Y.T., Gong W., Li J. (2023). A ratiometric fluorescence sensor for tetracycline detection based on two fluorophores derived from Partridge tea. Mikrochim. Acta..

[22] Dai Y., Zhang Y., Liao W., Wang W., Wu L. (2020). G-quadruplex specific thioflavin T-based label-free fluorescence aptasensor for rapid detection of tetracycline. Spectrochim. Acta. A Mol. Biomol. Spectrosc..

